# Fabrication of Sustainable Diatomite-Based Foams with a Micro-Macroporous Synergistic Structure

**DOI:** 10.3390/ma18091968

**Published:** 2025-04-26

**Authors:** Hailong Ning, Zhiwu Li, Ning Liu, Chengling Li, Yao Lu, Long Li

**Affiliations:** 1 School of Materials Science and Engineering, Shenyang University of Chemical Technology, Shenyang 110142, China; nhl2318@163.com; 2 Inner Mongolia Dongsheng Diatomite Science and Technology Innovation Industrial Park Co., Ltd., Ulanqab 013400, China; 3 Yingkou Dongsheng Industrial Co., Ltd., Yingkou 115000, China

**Keywords:** diatomite, multistage aperture, chemical foaming, functional materials, microporous macropores, humidity control performance

## Abstract

This study developed a foamed material with a synergistic microporous-macroporous structure through chemical foaming and high-pressure curing to better utilize the microporous properties of diatomaceous earth in building materials. The effects of different amounts of foaming agent, foam stabilizer, and CaO/SiO_2_ on the mechanical properties and pore structure of the samples were investigated. The experimental results demonstrate that, under the influence of the foaming agent, the foam material has developed a multi-stage pore structure that integrates both macropores and micropores. This unique structure results in a dry density range of 467–670 kg/m^3^, thereby achieving significant material lightweighting. In addition, these macropores enhance the interaction between the micropores of diatomaceous earth and the external environment interface, thereby achieving a balance between the material’s structural stability and functional properties. The material exhibits a porosity of 76.9% and a specific surface area of 42.9 m^2^/g, while maintaining a high compressive strength of 2.67 MPa. This work provides a technological pathway for the fabrication of multifunctional building materials that have both lightweight and eco-functional properties.

## 1. Introduction

As the cornerstone of the global construction industry, the large-scale use of cement, while promoting urbanization, has also led to a serious carbon emission problem. According to statistics, cement production contributes about 8% of global carbon dioxide emissions, becoming the second-largest industrial source of carbon emissions after the energy industry [1,2]. Against the backdrop of the “double carbon” target, the construction industry faces an urgent imperative to develop sustainable alternatives that maintain structural functionality while reducing ecological footprints. The persistent dominance of cement-based materials in construction applications, particularly within traditional wall material systems, raises noteworthy environmental concerns due to their substantial ecological footprint. Current wall material technologies continue to present functional limitations, despite market prevalence. Conventional masonry systems exhibit three prominent disadvantages: elevated resource intensity during production, labor-intensive installation processes, and excessive weight characteristics that complicate structural engineering requirements. While lightweight partition panels demonstrate improved constructability through modular assembly, their technical performance reveals critical deficiencies. Three core challenges persist: insufficient acoustic attenuation capabilities, compromised material durability under cyclic environmental exposure, and dimensional instability under hygrothermal stresses. Furthermore, certain composite formulations employed in panel manufacturing have been observed to emit volatile organic compounds (VOCs) and particulate matter during both the fabrication and service phases [3,4,5]. These emissions present dual risks of environmental contamination and potential human health impacts through inhalation pathways or bioaccumulation mechanisms. These problems indicate that optimizing existing wall materials or developing new, environmentally friendly, high-performance materials has become an important research direction in the construction industry [6,7,8]. Foamed geopolymer, a lightweight porous inorganic thermal insulation material, demonstrates significant potential for reducing building energy consumption, structural loads, and transportation/construction costs [9,10,11,12]. Its notable advantages—including superior thermal insulation, environmental sustainability [13], acoustic performance [14], fire resistance, and seismic enhancement—have facilitated widespread adoption in construction [15,16,17]. Current research focuses on enhancing its eco-efficiency and functionality through alternative raw materials, such as fly ash [18,19,20], metakaolin [21,22,23], and blast furnace slag [24,25,26], aiming to develop advanced green lightweight composites.

Diatomite, a biogenic siliceous sedimentary rock formed from fossilized diatoms and microorganisms [27], boasts abundant global reserves and distinct physicochemical properties. Composed primarily of amorphous silica with pozzolanic activity, its natural porous structure confers exceptional characteristics, including adsorption capacity [28,29,30], thermal insulation [31,32,33], sound absorption [34], and humidity regulation [35]. These attributes have spurred extensive investigations into its construction applications. Hassan et al. [36] developed a cement mixture with a 28-day compressive strength of 62.9 MPa by substituting 10% of the cement with diatomite activated by NaOH, effectively reducing cement consumption without compromising strength. Ouyang et al. [37] demonstrated that diatomite-incorporated cement paste achieved a compressive strength comparable to that of pure cement through optimized hydration processes and microstructural development. He et al. [38] observed that diatomite substitution for fly ash in lightweight engineered cementitious composites (LECC) reduced hydration degree and increased porosity, thereby decreasing compressive and tensile strength. However, supplementary water addition enhanced hydration product formation and densified the D-LECC matrix, ultimately improving compressive performance.

This study aims to develop a new type of diatomaceous earth-based foam material with a strong structure and diverse functions by chemical foaming and high-pressure curing techniques. The synergistic effects of blowing agent concentration, stabilizer dosage, and CaO/SiO_2_ ratio on the mechanical properties of the material and the integration of microcellular functionality were systematically investigated. This is of great significance for the current global goal of achieving energy saving and emission reduction, promoting the functionalization of building materials, and sustainable development.

## 2. Materials and Methods

### 2.1. Materials and Foam Preparation Process

The materials were fabricated through chemical foaming and autoclaved curing processes. The diatomite (with its main components detailed in Table 1), silica fume (>96%), aluminum powder for foaming, and polycarboxylate superplasticizer were sourced from Inner Mongolia Dongsheng Diatomite Technology Innovation Industrial Park Co., Ltd. (Ulanqab, China). The remaining experimental materials include calcium oxide (CaO, >99%) and calcium stearate (C_36_H_70_CaO_4_, >99%) were purchased from Aladdin Biotechnology Ltd. (Shanghai, China).

Diatomite, calcium oxide, silica fume, and calcium stearate were weighed according to predetermined ratios and homogenized in a mixer. An optimized mixture of water and superplasticizer was then added, followed by low-speed mixing at 300 rpm for 5 min to achieve a uniform slurry. The foaming agent was incorporated under high-speed mixing at 1200 rpm for 30 s to ensure controlled bubble formation. The foamed slurry was immediately transferred to molds and allowed to stabilize at room temperature. The molds were cured in a forced-air drying oven at 60 °C for 6 h before demolding. The specimens were subsequently treated in an autoclave at 180 °C under 1 MPa saturated steam pressure for 5 h, followed by gradual cooling to room temperature to complete the synthesis process.

### 2.2. Mix Design

Dry density and compressive strength are critical performance metrics for foamed materials. In practical applications, the density of foamed materials typically ranges from 300 to 1800 kg/m^3^ [39]. While density significantly influences strength [40], the CaO/SiO_2_ ratio within the material system also plays a pivotal role in strength development [41,42]. In this study, under fixed conditions of 10% silica fume, 0.3% superplasticizer, and a liquid-to-solid ratio of 0.55, with autoclave curing at 180 °C and 1 MPa for 5 h, the effects of varying foaming agent content (0.1–0.5%), foam stabilizer dosage (0.4–2.0%), and CaO/SiO_2_ ratios (0.4–1.2) on the physical properties and pore structure of foamed materials were systematically investigated. The mix design parameters for the foamed materials are detailed in Table 2.

### 2.3. Characterization

#### 2.3.1. Dry Density

The dry density of the foamed material was measured using the mass-volume method. First, the samples that had been cured were cut to the dimensions of 40 mm × 40 mm × 40 mm. The cut samples were then placed in an oven at (105 ± 5) °C for 24 h to ensure that they dried to a constant weight. After the samples were cooled to room temperature, they were weighed using an electronic balance with an accuracy of 0.1 g. The length, width, and height of the samples were measured using vernier calipers with an accuracy of 0.1 mm, and the dry density of the samples was then calculated by the mass-volume method. Three samples were measured in each group, and the average value was taken as the final result.

#### 2.3.2. Compressive Strength

The compressive strength of the specimens was measured according to ASTM C495-2022 [43]. Before conducting the tests, the specimens were cut into 40 mm cubes, smoothed with sandpaper, and dried at (105 ± 5) °C for 24 h. Compressive strength tests were conducted using a DYE-300S servo-hydraulic system (Haichuang Instrumentation Franchise, Hebei, China) at a stress rate of 0.5 MPa/s (0.48 mm/min for a 40 mm specimen) until failure. Three replicates per group were tested, and results were averaged.

#### 2.3.3. Phase Analysis

Phase identifications were performed using X-ray diffraction (XRD, Bruker D8 instruments, Billerica, MA, USA) with nickel-filtered CuKα radiation at 30 kV and 20 mA. The XRD tests were performed at a 2θ range of 5–80° with a scanning rate of 2°/min.

#### 2.3.4. Pore Structure Analysis

**Figure 1:**
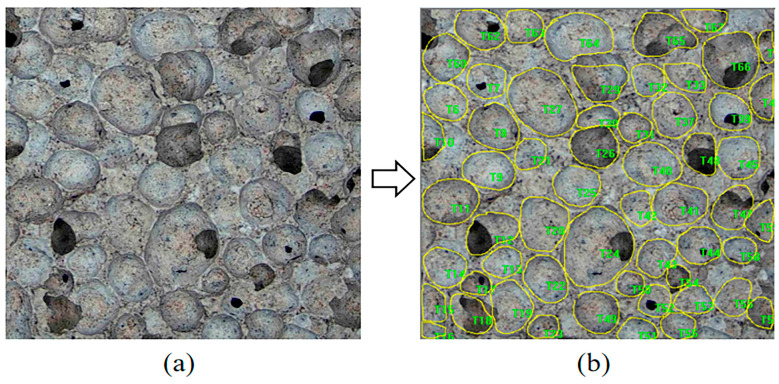
(**a**) Original image and (**b**) processed image of the sample.

For pore parameter quantification, an image analysis protocol was implemented. Specimens were first dried in an oven at 105 °C for 6 h. Cross-sectional samples (40 mm × 40 mm × 40 mm) were then prepared by cutting parallel to the porous surface using precision blades, followed by polishing with abrasive paper (800 grit) to achieve flatness. Residual particles were removed via compressed air jetting. High-resolution imaging was performed using an industrial CCD microscope (Ke Zhongxing Flagship Store, Guangdong, China), as shown in Figure 1a. The acquired images were subjected to pore identification using Image-Pro Plus 6.0 software, as shown in Figure 1b, and the resulting parameters were used to calculate the porosity and pore size distribution. Three samples from each group were analyzed, and the final porosity and pore size distribution parameters were averaged from the results of the three samples.

#### 2.3.5. Surface Area Analysis and Porosity Testing

The specific surface area of the samples was analyzed using an ASAP 2460 low-temperature physical adsorption instrument (Mack Instruments, Georgia, USA). The samples were first vacuum-degassed at 150 °C for 6 h, followed by testing at liquid nitrogen temperature (−196 °C). The specific surface area was calculated by the BET method. The pore volume was determined by the adsorption amount at a relative pressure of 0.95 MPa. The pore size distribution was calculated using the BJH method.

#### 2.3.6. Moisturizing Performance Testing

The moisturizing properties of the samples were measured according to ISO 12571-2013 [44]. Before the start of the test, the specimens (100 mm × 100 mm × 10 mm) were pre-treated in a forced-air oven at (105 ± 5) °C for 24 h, until the mass change was less than 0.1%. Prepare two sealed glass enclosures large enough to hold the samples and adjust the room temperature so that the temperature of the two sealed environmental chambers is maintained at (23 ± 0.5) °C. Relative humidity of 98% was maintained by a saturated solution of potassium sulfate (K_2_SO_4_). A solution of magnesium chloride hexahydrate (MgCl_2_) was used to control 33% relative humidity. The RH inside the chamber was verified by a humidity sensor probe (RH accuracy ± 1). During the hygroscopic phase, the dried specimens were transferred to the chamber at 98% RH, and the mass of the specimens was recorded hourly for 24 h. During the dehumidification phase, the specimens were immediately transferred to a chamber at 33% RH, and the same measurement protocol was applied. Three samples were measured in each group, and the final results were averaged over the three samples.

(1) $$W_{t}=\frac{m_{t}-m_{0}}{m_{0}}\times 100\%$$

where *W_t_* is the moisture absorption/expulsion rate of the sample at time *t*, %; *m_t_*—mass of the sample at the moment *t*, g; *m*_0_—mass of the sample after complete drying, g.

## 3. Results and Discussion

### 3.1. Morphological and Porosity Analysis

**Figure 2:**
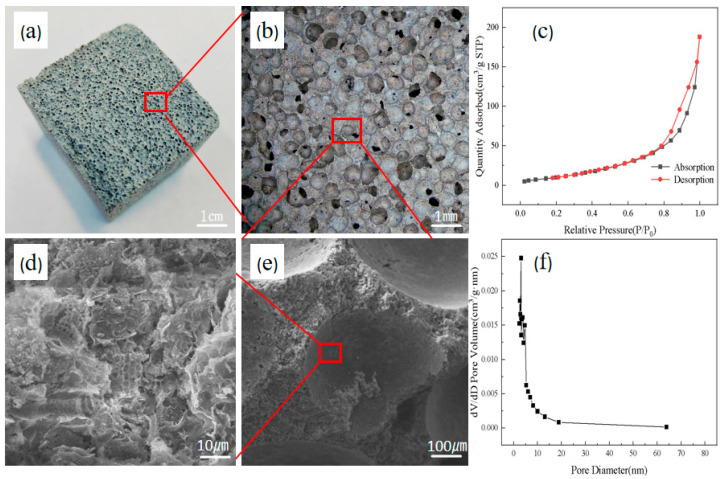
Photographs and SEM micrographs of foamed materials and BET analysis plots. (**a**) Sample normal shooting photos; (**b**) Photographs of samples taken at 40 times magnification; (**c**) N_2_ adsorption/desorption curves of samples; (**d**) SEM image of the sample magnified 1000 times; (**e**) SEM image of the sample magnified 100 times; (**f**) BJH desorption curves of samples.

Figure 2 illustrates the structural characteristics of the multi-stage pore foam material using sample C3, whose formula parameter located in the middle position is highly representative. In this figure, macroscopic imaging (Figure 2a,b) verifies a uniform void distribution, while SEM analysis (Figure 2d,e) exposes the synthetic architecture, where diatomite-derived micropores are uniformly distributed across the walls and interstices of macropores formed by the foaming agent. From the surface area analysis and porosity testing analysis of the samples, it can be seen that the presence of a large number of microporous structures in the material endows it with strong adsorption properties (Figure 2c,f), and the average pore size of most of these micropores is distributed between 2 and 5 nm. This dual-scale porosity ensures optimal micro-macro pore distribution, an optimized pore size gradient, and enhanced surface area exposure via diatomite’s microporous network. These features collectively amplify interfacial interactions between micropores and the external environment, thereby improving overall material performance. The resultant structure demonstrates a synergistic coexistence of macroporous and microporous systems, forming an effective hierarchical pore network.

### 3.2. Effect of Blowing Agent on Material Properties

**Figure 3:**
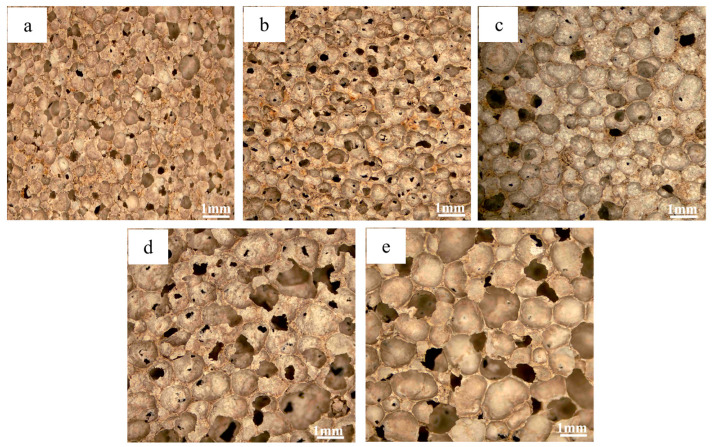
Micrographs of foamed materials with different amounts of blowing agent: (**a**) 0.1%; (**b**) 0.2%; (**c**) 0.3%; (**d**) 0.4%; (**e**) 0.5%.

**Figure 4:**
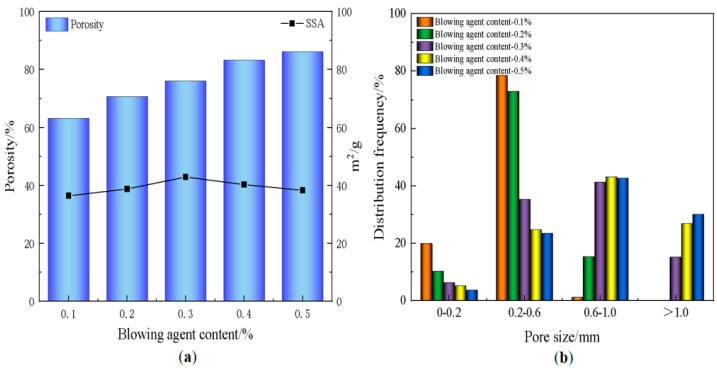
(**a**) Variation of porosity and specific surface area; (**b**) pore size distribution of foamed materials with different amounts of foaming agent.

The pore architecture in foamed materials is governed by the decomposition of blowing agents within the slurry, generating gas bubbles that become entrapped during matrix solidification, ultimately forming a porous structure. Consequently, the foaming agent dosage critically influences the material’s pore characteristics [45]. Figure 3 shows the photographs of the pore structure of the foamed material at different blowing agent dosages, from which it can be seen that an increase in blowing agent content leads to a gradual increase in pore size and a gradual thinning of the pore wall, which is due to the increase in the gas production, which promotes the coalescence of the bubbles in the confined slurry volume. This mechanism leads to a continuous increase in porosity. Figure 4 shows the trends of material porosity, specific surface area (SSA), and pore size distribution at different blowing agent dosages. As the blowing agent dosage increased from 0.1% to 0.5%, the porosity of the material rose continuously, and the average pore size also increased. Meanwhile, its specific surface area exhibited a trend of first increasing and then decreasing. When the blowing agent dosage reached 0.3%, pores larger than 1 mm began to emerge. The pore size distribution became more diverse, with the porosity at 76.1%. Additionally, the specific surface area achieved its maximum value of 42.9 m^2^/g. The optimal balance between structural regularity and functional performance was realized. As the blowing agent was further increased to 0.5%, the porosity was slightly increased, but the mechanical integrity was greatly reduced due to the thin wall. At the same time, too much blowing agent also leads to the merging of the original tiny pores in the material, resulting in a coarsening of the pore structure and a decrease in the specific surface area. Therefore, the 0.3% formulation was considered to be the functionally optimal configuration, which harmonized the refinement of the pore structure and the structural robustness.

**Figure 5:**
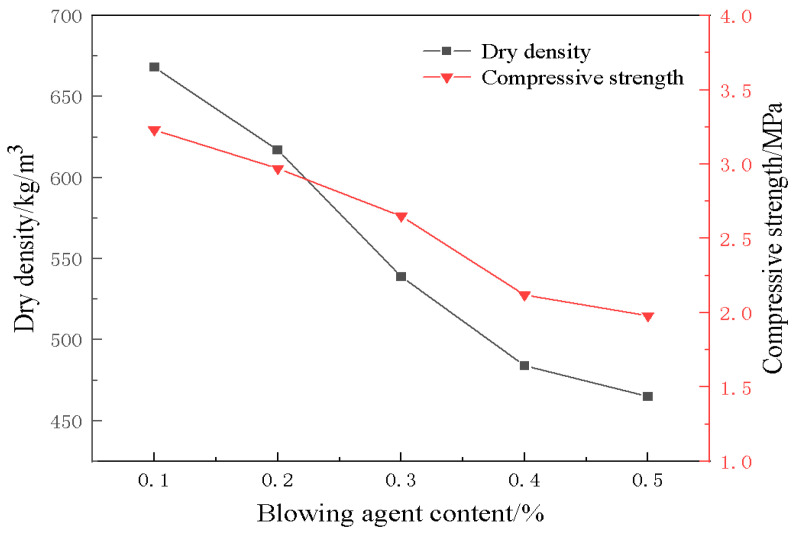
Effect of blowing agents on compressive strength and density of materials.

Figure 5 shows the trend of dry density and compressive strength of foamed materials with different blowing agent dosages. From the figure, it can be observed that the dry density of the material decreases gradually with an increase in the blowing agent dosage. This is mainly because, with an increase in blowing agent dosage, more bubbles can be generated to occupy the volume of the material, resulting in a decrease in the proportion of the solid phase of the material. The bubbles, as internal defects in the material, are prone to becoming stress concentration points, and cracks are more likely to appear at the interface of the bubbles when the material is subjected to compression. As a result, the compressive strength of the material also decreases. To ensure the material has a certain compressive strength, a foaming agent dosage of 0.3% is more reasonable; at this time, the dry density of the material is 539 kg/m^3^, with a compressive strength of 2.65 MPa.

**Figure 6:**
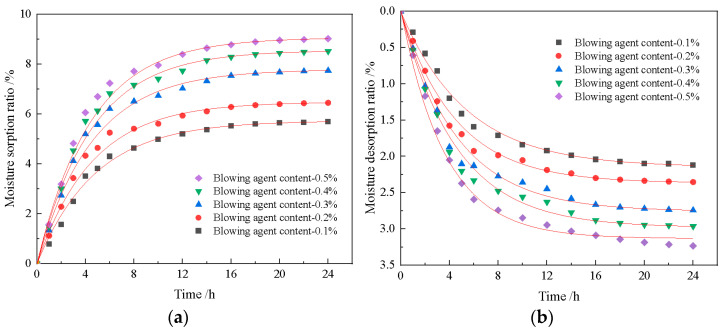
Moisturizing properties of materials with different amounts of blowing agents, (**a**) Moisture absorption curve; (**b**) Moisture release curve.

As demonstrated in Figure 6, the humidity regulation capacity of the foamed material improves progressively with increasing blowing agent content, directly correlating with enhanced porosity. This phenomenon arises from the hierarchical pore structure, where increased macroporosity facilitates environmental interaction with diatomite-derived micropores, thereby amplifying both moisture absorption and desorption rates. During the initial 24-h testing period, absorption/desorption rates exhibit rapid growth before stabilizing. Notably, formulations exceeding 0.2% foaming agent display significant humidity regulation improvements, with the 0.3% dosage achieving an optimal balance. At this moment, the peak moisture absorption is 8.51%, and the peak moisture desorption is 2.97%.

Beyond this threshold, while humidity regulation continues to improve marginally, mechanical performance degrades substantially due to excessive pore wall thinning. The 0.3% formulation thus represents the critical equilibrium point where hierarchical porosity maximizes functional performance without compromising structural integrity, validating the effectiveness of multiple pore engineering in hygroscopic material design.

### 3.3. Effect of Foam Stabilizer on Material Properties

**Figure 7:**
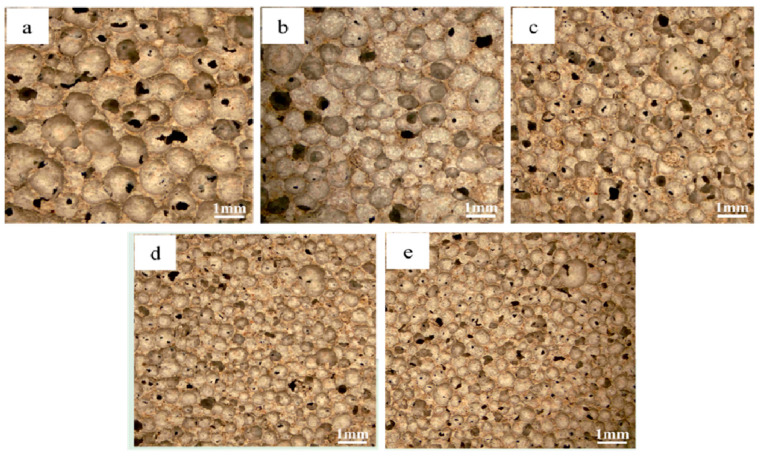
Micrographs of foamed materials with different amounts of foam stabilizer: (**a**) 0.4%; (**b**) 0.8%; (**c**) 1.2%; (**d**) 1.6%; (**e**) 2.0%.

**Figure 8:**
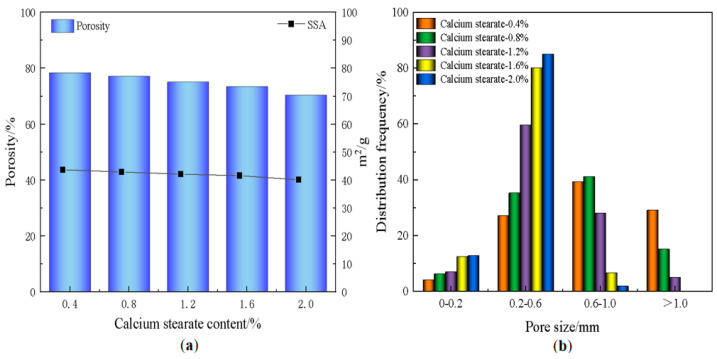
(**a**) Variation of porosity and specific surface area; (**b**) pore size distribution of foamed materials with different amounts of foam stabilizer.

Figure 7 shows the photos of the pore structure of the foamed materials with different amounts of stabilizer, from which it can be observed that the increase of the stabilizer content leads to a gradual decrease in the pore size of the materials. As illustrated in Figure 8, the porosity and specific surface area of the material decrease with the increasing amount of the stabilizer, calcium stearate, while the average pore size also diminishes. This is because calcium stearate can increase the mechanical strength of the bubbles, effectively limiting their coalescence [46], thus reducing the proportion of the gas phase in the system. At a foam stabilizer dosage of 0.4%, the limited interfacial modification produced negligible stabilization effects. Foam stabilizer doping of 0.8% significantly refines the pore size while increasing the pore wall thickness, but an excess of foam stabilizer leads to stabilization saturation with minimal further structural changes. After functional saturation, overdosing disrupts the rheology of the slurry, affecting foam expansion and further reducing porosity [47]. This dual mechanism—pore stabilization versus rheological compromise—highlights the critical balance required in stabilizer optimization, with moderate dosages achieving optimal pore uniformity while preserving material processability.

**Figure 9:**
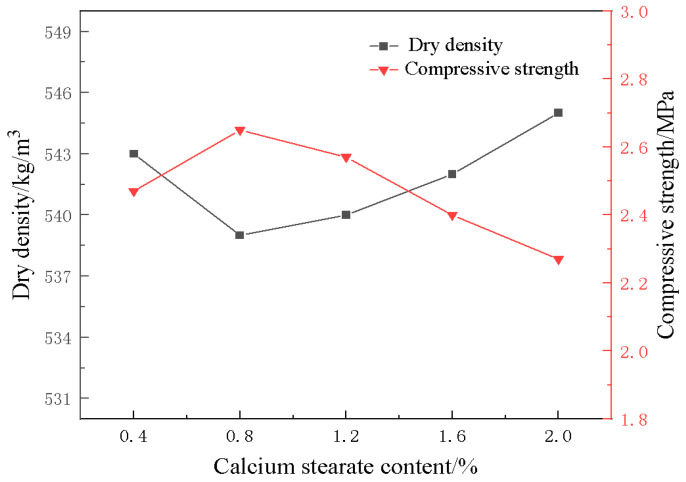
Effect of foam stabilizers on compressive strength and density of materials.

The trends of dry density and compressive strength of the foamed materials under different amounts of foam stabilizer are presented in Figure 9. From the figure, it can be seen that with an increase in the dosage of foam stabilizer calcium stearate, the dry density of the foamed material does not change much, while the compressive strength shows a trend of increasing first and then decreasing. The compressive strength reaches its maximum when the dosage of foam stabilizer is 0.8%, which is 2.65 MPa. This may be because the appropriate amount of foam stabilizer can improve the stability of the bubbles and reduce the rupture and overflow of the bubbles, while excessive foam stabilizer increases the viscosity of the slurry and affects the cross-linking of the polymer matrix, which results in a decrease in the compressive strength of the material.

### 3.4. Effect of CaO/SiO_2_ on Material Properties

**Figure 10:**
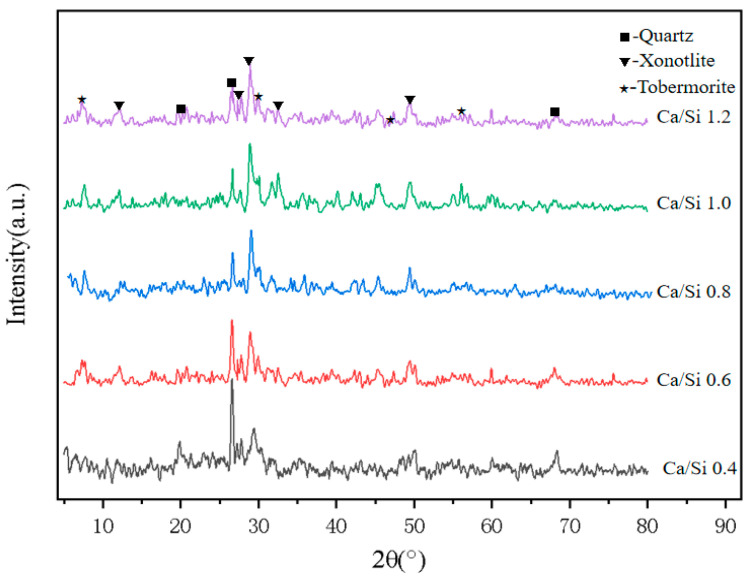
Physical phase composition of foams with different CaO/SiO_2_ ratios.

To clarify the effect of the calcium-silicon ratio on the hydration products, we performed XRD analysis on samples (C1–C5) with different CaO/SiO_2_ ratios (Figure 10). As depicted in the figure, the primary hydration products formed during the high-pressure curing process were tobermorite (Ca_5_Si_6_O_16_(OH)_2_·4H_2_O) and xonotlite (Ca_6_Si_6_O_17_(OH)_2_). These cementitious phases are widely acknowledged for their significant contributions to the mechanical durability of the composite, thanks to their interlocking crystal structures. As the CaO/SiO_2_ ratio increased from 0.4 to 0.8, the diffraction peaks of quartz were notably weakened. Meanwhile, the characteristic peaks of the hydration products tobermorite and xonotlite exhibited a marked increase in intensity. This suggests that the addition of calcium oxide can accelerate the dissolution of siliceous raw materials, thereby promoting the hydration reaction within the system. With the increase of CaO/SiO_2_ ratio above 0.8, the diffraction peaks of both hydration products and quartz no longer change significantly at this time, indicating that the alkali excitation played by calcium oxide has reached its limit, and the failure of excess CaO to fully participate in the hydration reaction may negatively affect the intensity.

**Figure 11:**
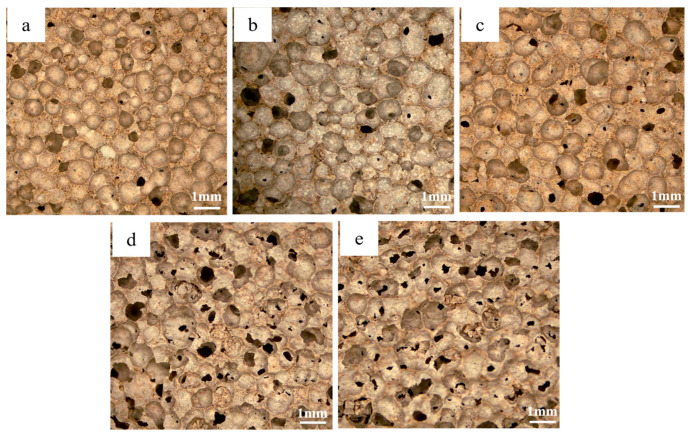
Micrographs of foamed materials with different CaO/SiO_2_; (**a**) C/S0.4; (**b**) C/S0.6; (**c**) C/S0.8; (**d**) C/S1.0; (**e**) C/S1.2.

**Figure 12:**
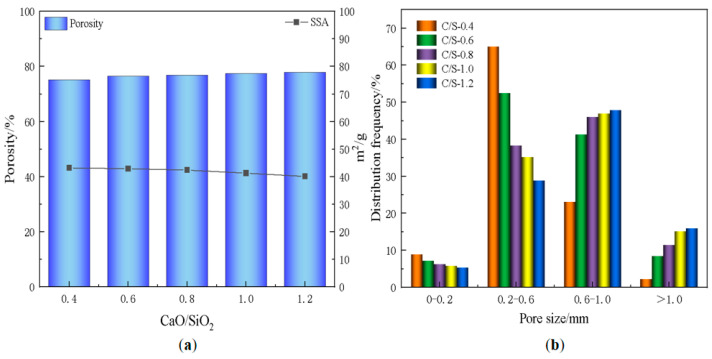
(**a**) Variation of porosity and specific surface area; (**b**) pore size distribution of foamed materials with different CaO/SiO_2_.

Figure 11 demonstrates the variation of the pore structure of the foamed materials at different CaO/SiO_2_ ratios. At CaO/SiO_2_ ratios of 0.4–0.8, the pore wall integrity of the foamed material is better, with very few interconnected pores, which is attributed to the fine particles of diatomite and its inherent microporous structure. This unique structure allows diatomaceous earth particles to adhere to the surface of the foam through interfacial interactions, thus increasing the stability of the foam. When the CaO/SiO_2_ ratio is increased to 1.0, the increase in CaO content accelerates the gas that is produced by the exothermic reaction of the blowing agent during the hydration process. This promotes the rapid expansion of gas bubbles, leading to an increase in pore size and a decrease in pore wall integrity. It can also be seen from Figure 12 that the porosity and average pore diameter of the material tend to increase with the increase of CaO/SiO_2_. However, the reduction in the proportion of diatomite within the material led to a slight decrease in its specific surface area. When the CaO/SiO_2_ ratio was 0.8, the material achieved a porosity of 76.9% and a specific surface area of 42.4 m^2^/g, while maintaining an excellent pore.

**Figure 13:**
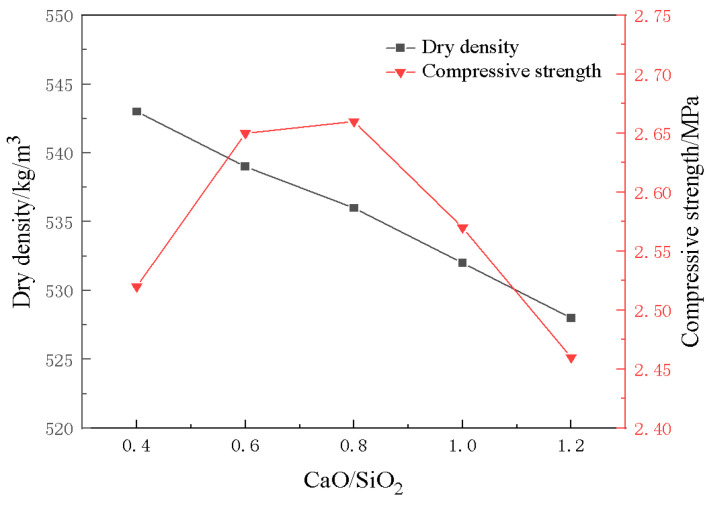
Effect of CaO/SiO_2_ on compressive strength and density of materials.

Figure 13 illustrates the trends of dry density and compressive strength of foamed materials under varying CaO/SiO_2_ ratios. From the figure, it can be seen that with an increase in CaO/SiO_2_ from 0.4 to 1.2, the compressive strength of the foamed material shows a trend of increasing first and then decreasing. The compressive strength reaches its maximum when CaO/SiO_2_ is 0.8. This may be because the relative content of CaO in the system increases with an increase in CaO/SiO_2_, and the hydration reaction becomes more and more adequate. At the same time, the increased alkalinity of the system also promotes the decomposition rate of the aluminum powder, resulting in a continued increase in the compressive strength of the material and a gradual decrease in density. However, a CaO/SiO_2_ ratio that is too high may cause CaO to exist in a free state, which triggers microcracks and weakens the interfacial bonding, resulting in reduced strength.

## 4. Conclusions

This study fabricated a foam material with a multistage pore size structure by combining a natural microporous material (diatomaceous earth) with an engineered macroporous framework. This multistage pore size structure effectively balances functional properties such as moisture control and adsorption with structural reliability.

(1) The blowing agent dosage has a large effect on the porosity, with the increase of blowing agent dosage, the porosity and average pore diameter of the foamed material increase, the specific surface area shows a trend of rising and then decreasing, and the dry density and compressive strength are gradually decreasing. When the dosage of the blowing agent is 0.3%, the specific surface area of the foamed material reaches a maximum of 42.9 m^2^/g, the dry density is 539 kg/m^3^, and the compressive strength is 2.65 MPa.
(2) With the addition of foam stabilizer calcium stearate, the compressive strength of the foamed material decreases and then increases, and when the amount of calcium stearate added is 0.8%, it can obviously improve the pore structure and compressive strength of the foamed material.
(3) The construction of multilevel pore size is conducive to the advantage of diatomite microporosity. With an increase in foaming agent dosage, the porosity of the material increases continuously, and the moisture regulating performance also improves. When the dosage of the blowing agent is 0.3%, the dosage of the foam stabilizer is 0.8%, and the CaO/SiO_2_ is 0.8, the comprehensive performance of the foamed material is the best. At this time, the porosity of the foamed material was 76.9%, the maximum moisture absorption rate was 8.51%, the maximum moisture release rate was 2.97%, and the compressive strength reached 2.67 MPa.

This type of foam material, characterized by its multistage pore size, exhibits properties such as lightweight, adsorption capacity, and environmental friendliness. It holds broad application prospects in the construction field.

## Figures and Tables

**Table 1:** Main chemical composition of diatomite (%).

Composition	SiO_2_	Al_2_O_3_	Fe_2_O_3_	CaO	K_2_O	MgO	Na_2_O	LOI
Mass fraction	56.02	12.04	7.76	1.69	1.50	1.61	0.60	16.17

**Table 2:** Mix the proportion of foamed material.

Code	Al (g)	CS (g)	Diatomite (g)	CaO (g)	Silica Fume (g)	PCE (g)	Water (g)
A1	0.2	1.6	124	56	20	0.6	110
A2	0.4	1.6	124	56	20	0.6	110
A3	0.6	1.6	124	56	20	0.6	110
A4	0.8	1.6	124	56	20	0.6	110
A5	1.0	1.6	124	56	20	0.6	110
B1	0.6	0.8	124	56	20	0.6	110
B2	0.6	1.6	124	56	20	0.6	110
B3	0.6	2.4	124	56	20	0.6	110
B4	0.6	3.2	124	56	20	0.6	110
B5	0.6	4.0	124	56	20	0.6	110
C1	0.6	1.6	140	40	20	0.6	110
C2	0.6	1.6	124	56	20	0.6	110
C3	0.6	1.6	108	72	20	0.6	110
C4	0.6	1.6	93	87	20	0.6	110
C5	0.6	1.6	80	150	20	0.6	110

## Data Availability

The original contributions presented in this study are included in the article. Further inquiries can be directed to the corresponding authors.
